# “Bud to bloom”—hormonal coordination in floral initiation

**DOI:** 10.1111/plb.70089

**Published:** 2025-08-25

**Authors:** R. Baral, A. Vainer, S. Melzer, B. Hause, S. Panda

**Affiliations:** ^1^ Department of Cell and Metabolic Biology Leibniz Institute of Plant Biochemistry Halle (Saale) Germany; ^2^ Department of Plant and Environmental Sciences Weizmann Institute of Science Rehovot Israel; ^3^ Gilat Research Center Agricultural Research Organization Rural delivery Negev 85280 Israel; ^4^ Botanical Institute Christian‐Albrechts University of Kiel Kiel Germany

**Keywords:** Abscisic acid, *Arabidopsis*, auxin, brassinosteroids, crosstalk, cytokinin, ethylene, floral organ, flowering regulatory network, flowering time, gibberellic acid, jasmonic acid, salicylic acid, shoot apical meristem, tomato

## Abstract

Hormones have a dominant role in shaping the destiny of plant reproduction. Recent breakthroughs in our understanding of hormone function during floral development have revealed the pivotal roles of cytokinin, gibberellin and auxin. Cytokinin and gibberellin regulate the size and coordination of floral meristems, while auxin and cytokinin take centre stage in initiating and developing organs. In the past decade, remarkable insights have emerged, revealing the dynamic nature of hormonal impacts throughout reproductive development. It has become evident that a complex network, involving multiple plant hormones, orchestrates the success of plant reproduction. Despite substantial cover of certain aspects of plant reproductive development, there remain significant gaps in our understanding of hormonal regulation and the intricate crosstalk between hormones. In this comprehensive review, we delve into current knowledge and address lingering questions regarding hormone‐mediated flower development. By arming ourselves with this knowledge, we pave the way for innovative strategies in effective fruit set management and crop improvement.

## Graphical abstract

This review unpacks hormonal networks in flower development, revealing how ‘bud to bloom’ transitions shape yield and plant resilience.

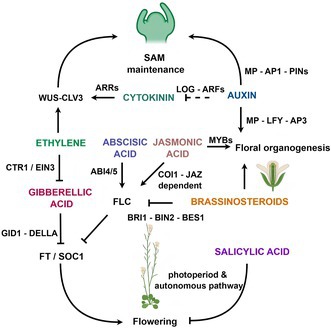

## INTRODUCTION

Improvements in agronomic and nutritional features necessitate a thorough understanding of the major molecular factors involved in flower and fruit development. High throughput sequencing technologies providing high‐resolution spatiotemporal gene expression data, together with mutant analysis and hormone profiling have been major factors behind advances in dissecting the molecular pathways of these fundamental processes (Meir *et al*. 2021). The coordinated development of floral organs is essential for successful reproduction in flowering plants, ultimately enabling fertilization and fruit formation. In monocots, such as rice and maize, hormone‐mediated reproductive development has been extensively studied (Cao & Chen 1995; Cao & Shannon 1997; Yoshida & Nagato 2011; Huang *et al*. 2018), while *Arabidopsis thaliana* and tomato (*Solanum lycopersicum*), are dicots, in which hormone the roles of flower and fruit development have been explored (Chandler & Werr 2020; Fenn & Giovannoni 2021).

Floral organogenesis is orchestrated through a series of morphogenetic events, primarily involving stem cell proliferation and expansion at the shoot apical meristem (SAM), and is governed by complex regulatory networks, comprising phytohormone signalling pathways, transcription regulators and epigenetic modifications (Jack 2004; Chen *et al*. 2018; Xu *et al*. 2021; Ramos‐Pulido & de Folter 2023). In higher plants, SAM undergoes distinct developmental transitions, giving rise to diverse lateral organs, such as leaves, inflorescence and flowers, depending on the development stage of the plant (Barton 2010). In shoots, SAM is a dome‐shaped, dynamic, specialized structure containing three distinct functional domains – central zone (CZ), organizing centre and rib zone, which together constitute a stem cell niche and are maintained in a pluripotent state by signals from adjoining cells (Steeves & Sussex 1989). The characterization of each functional domain of a stem cell niche and the organization of SAM has been well described (Ha *et al*. 2010). The dynamic changes in cellular states define the fate of organogenesis and initiate from rapidly dividing daughter cells displaced from CZ to the peripheral zone at SAM. The timing of floral initiation, or the transition from vegetative to reproductive development, is influenced by environmental cues, such as temperature, photoperiod and endogenous stimuli. The floral transition encompasses conversion of SAM into an inflorescence meristem (IM) and subsequent formation of flowers. The genetic regulatory network channels both interior and external environmental signals to coordinate and activate floral meristem (FM) identity genes, to give rise to FMs (or floral primordia) from regions of the IM. After initiation of the FM, the fate of four different types of floral whorls is specified by the respective activation of floral organ identity genes encoding MADS domain transcription factors (Lohmann & Weigel 2002). The above results laid the foundations for the well‐known ABC genetic model of floral organ identify specification (Schwarz‐Sommer *et al*. 1990; Bowman *et al*. 1991). According to the ABC model in *Arabidopsis*, sepal formation is regulated by *APETALA1* (*AP1*) and *APETALA2* (*AP2*), which are classified as A‐class genes. The combined action of A‐ and B‐class genes – *APETALA3* (*AP3*) and *PISTILLATA* (*PI*) – determines petal development, whereas combined activities of B‐ and C‐class genes – *AGAMOUS* (*AG*) – control stamen development, while carpel development is solely controlled by C‐class genes. The identification of four *SEPALLATA* genes (*SEP1–SEP4*), which function redundantly with previously identified ABC class genes, led to refinement of the model into the ABCE framework of flower development (Causier *et al*. 2010; Chanderbali *et al*. 2016; Irish 2017). The ABCE model has been extended to monocot flowers, despite the morphological differences between eudicot and monocot floral structures. There is substantial evidence supporting the conservation of homeotic genes across vascular plants, including homologues of*AP3* identified in monocots and in gymnosperms (Ambrose *et al*. 2000; Kanno *et al*. 2003; Whipple *et al*. 2004). Comparative analyses have underscored the functional conservation, evolutionary diversification, and progressive refinement of the ABC/ABCE model across angiosperm lineages, including both dicots and monocots (Soltis *et al*. 2007; Bowman *et al*. 2012; Chanderbali *et al*. 2016; Irish 2017; Bowman & Moyroud 2024). Moreover, the transformation of leaves into petal‐like structures and *vice versa* through constitutive overexpression of *SEP3* in combination with A‐ and B‐class genes (Pelaz *et al*. 2001), and conversely through mutation in *SEP* genes (Ditta *et al*. 2004), suggests a core developmental programme shared by floral organs and leaves, likely derived from a common ancestral organ (Sablowski 2010, 2015). This is further substantiated by evidence that components of the AG‐SEP‐AP3‐PI regulatory complexes can regulate genes involved in vegetative development, suggesting that floral identity proteins can also modulate the function of general developmental regulators (Sablowski 2010, 2015).

Previous studies have revealed that development of floral organs, (*viz*. sepal, petal, stamen and carpel) is to a large extent controlled by hormones, including, auxin, gibberellins (GA), cytokinin (CK), ethylene, abscisic acid (ABA), jasmonic acid (JA), salicylic acid (SA) and brassinosteroids (BR) (Martínez *et al*. 2004; Davis 2009; Campos‐Rivero *et al*. 2017; Ito *et al*. 2018; Schubert *et al*. 2019; Wybouw & De Rybel 2019; Li & He 2020; Cucinotta *et al*. 2021; Luo & Liu 2022; Zhao *et al*. 2022). This review aims to underpin the role and molecular networks of individual hormones as well as their crosstalk during flowering, including SAM maintenance, floral transition, floral organogenesis and development, as well as flowering time regulation, primarily focusing on model plants – *Arabidopsis* and tomato – but also highlighting important insights from monocot crops, such as rice and maize.

## CYTOKININ (CK) REGULATES SAM HOMEOSTASIS AND FLORAL ORGANOGENESIS

**Fig. 1:**
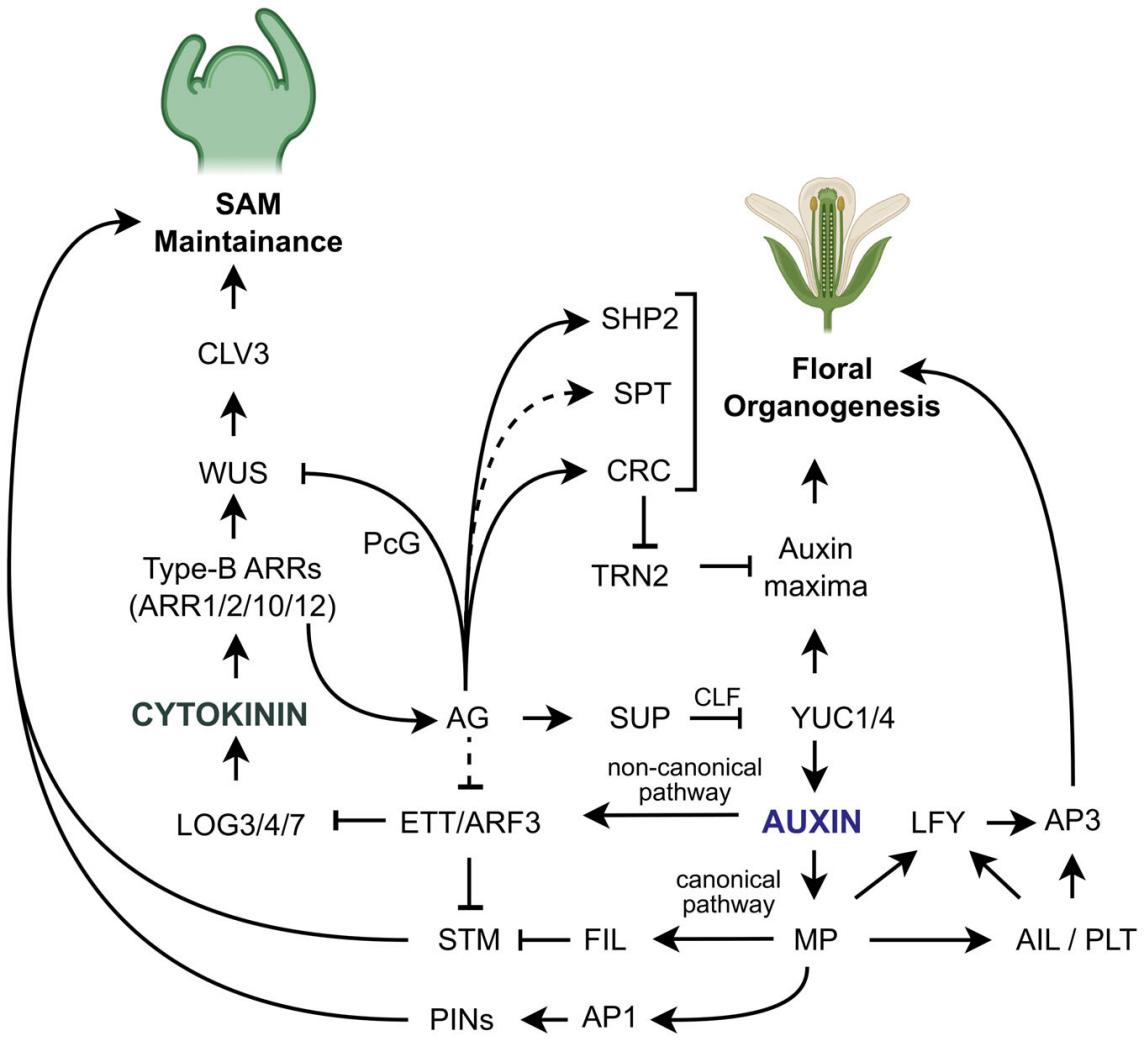
Crosstalk between auxin and cytokinin (CK) metabolism pathways and their roles in flower development. CLAVATA(CLV3)‐WUSCHEL(WUS) acts as a central hub to determine fate of the SHOOT APICAL MERISTEM (SAM) acquisition. The central regulator (CLV3‐WUS) is directly activated through primary CK signalling components such as type‐B ARABIDOPSIS RESPONSE REGULATORS (ARRs) and/or *via* AGAMOUS (AG)‐dependent manner. AG induces expression of carpel development TFs such as CRABS CLAW (CRC), SHATTERPROOF2 (SHP2) and SPATULA (SPT) to regulate floral organogenesis in a CK‐dependent manner. Furthermore, AG activates boundary gene *SUPERMAN* (*SUP*) that interacts with a Polycomb group (PcG) component; CURLY LEAF (CLF) and represses the auxin biosynthesis genes *YUCCA1* and *4* (*YUC1/4*), thus supporting interaction of auxin and CK signalling pathways in regulation of floral organogenesis. Likewise, CRC, a direct target of AG, supresses the expression of *TORNADDO2* (*TRN2*), which controls auxin homeostasis to reduce floral meristem activity for subsequent gynoecium development. The central TF MONOPTEROS (MP) of the auxin response pathway directly activates *AINTEGUMENTA‐LIKE*/*PLETHORA* (*AIL*/*PLT*) to promote the flower primordia identity gene APELATA3 (AP3) along with APETALA1, the TF that directly activates PIN‐FORMED (PINs) to regulate SAM homeostasis. Additionally, MP activates floral organ identity gene *LEAFY* (*LFY*) to regulate floral organogenesis. Furthermore, in non‐canonical auxin response pathway, ETTIN, also known as AUXIN RESPONSE FACTOR3 (ARF3), suppresses expression of *SHOOT MERISTMLESS* (*STM*) to enable floral meristem fate acquisition. Additionally, ETTIN represses the expression of CK biosynthetic genes, such as *LONELY GUY3/4/7*, supporting integration of CK and auxin in SAM homeostasis. Activation is indicated with a ‘sharp arrow’ (→), while inhibition is indicated with a ‘blunt arrow’ (┴). Broken lines indicate indirect manipulation of a process or unknown mechanism. Images were created with BioRender.com.

Cytokinin is an adenine‐derived phytohormone that positively regulates cell proliferation and is essential for SAM function through coordinated control of its biosynthesis, activation and cellular response. In *Arabidopsis*, an autoregulatory negative feedback loop CLAVATA (CLV)‐WUSCHEL (WUS) has been described as the central regulatory pathway of SAM homeostasis. Stem cells at the SAM release the CLAVATA3/EMBRYO SURROUNDING REGION (CLE) peptide CLV3, which is bound by the leucine‐rich repeat receptor‐like kinases (LRR‐RLKs) CLV1, CLV2 and RECEPTOR‐LIKE PROTEIN KINASE2 (RPK2), to supress transcription of the stem cell organizing gene, *WUS*, which limits the size of the stem cell pool. A reduced number of initial cells, in turn, will lead to reduced expression of *CLV3* that will increase *WUS* expression and the number of initial cells, thereby again increasing *CLV3* expression. In *Arabidopsis*, this SAM homeostasis is regulated by CK, by influencing meristem proliferation through the CLV peptide‐receptor complex (Lindsay *et al*. 2006). CK response is primarily mediated through four type‐B ARABIDOPSIS RESPONSE REGULATOR (ARR) transcription factors (ARR1, ARR2, ARR10, ARR12), and activates *WUS* transcription (Ferreira & Kieber 2005; Meng *et al*. 2017; Wang *et al*. 2017; Zubo *et al*. 2017) (Fig. 1). Conversely, type‐A ARRs regulate CK signalling through a negative feedback loop and negatively impact SAM size. *HAIRY MERISTEM* (*HAM*) genes are another key transcription regulator that interacts with WUS to coordinately control the stem cell niche and maintain SAM in various species, including *Arabidopsis* and tomato (Stuurman *et al*. 2002; Schulze *et al*. 2010; Engstrom *et al*. 2011; David‐Schwartz *et al*. 2013; Zhou *et al*. 2015; Hendelman *et al*. 2016). In tomato, silencing highly expressed *SlHAMs* at SAM and FM leads to over‐proliferation of cells in the peripheral regions of the SAM, driven by misexpression of the stem cell regulator WUS. Notably, reduced CK levels at *SlHAM*‐silenced leaves completely suppresses the over‐proliferation phenotype, indicating the regulatory link between *SlHAMs* and CK in meristem maintenance, and clarifies the additional role of *SlHAMs* in leaf morphogenesis (Hendelman *et al*. 2016). Similarly, the CK biosynthetic gene *LONELY GUY* (*LOG*) is expressed in SAMs and maintains meristematic cell fates in rice (Kurakawa *et al*. 2007). The dysfunction of *LOG* results in premature SAM termination due to low levels of active CK in the SAM. Furthermore, in rice, the zinc finger transcription factor (TF) DROUGHT AND SALT TOLERANCE maintains CK homeostasis at the reproductive SAM through direct activation of *OsCKX2* (*CYTOKININ OXIDASE/DEHYDROGENASE2*) encoding a CK‐degrading enzyme (Li *et al*. 2013).

Reproductive development in *Arabidopsis* starts with SAM transformation into an inflorescence meristem (IM), which develops the inflorescence with floral primordia that signify inception points for floral organogenesis, and ultimately forms four whorls of floral organs, namely sepals, petals, stamens and carpels. The size and shape of the IM is highly dependent on development stage and environmental cues that finally regulate cell division (Gaillochet *et al*. 2017; Jones *et al*. 2017). Transgenic *Arabidopsis* plants over‐expressing CK biosynthesis catalysing enzyme‐encoding gene *ARABIDOPSIS ATP/ADP ISOPENTENYLTRANS‐FERASE4* (*AtIPT4*) driven by an *APETALA1* (*AP1*) promoter result in increased size of the IM and number of floral primordia (Li *et al*. 2010). Overproduction of CK alters flower development, leading to an increased number of flowers. However, this also induces abnormal floral morphology, primarily due to the mis‐expression of organ boundary formation TFs, *CUP SHAPED COTYLEDON2* and *3* (*CUC2* and *CUC3*), which is mediated by CK signalling *ARABIDOPSIS HISTIDINE KINASE* (*AHK2* and *AHK3*) receptors (Li, Li, *et al*. 2010; Li, Su, *et al*. 2010). Recent work by Gómez‐Felipe *et al*. (2021) has elucidated that CKs induce carpel‐like structures in an AG‐dependent manner. AG, a MADS‐box TF, plays a key role in regulating the development of reproductive organs. In response to CK signalling, AG activates expression of carpel‐development TFs, such as *CRABS CLAW* (*CRC*), *SHATTERPROOF2* (*SHP2*) and *SPATULA* (*SPT*) (Fig. 1).

## THE ROLE OF AUXIN IN MERISTEM DEVELOPMENT AND DIFFERENTIATION

In recent decades, numerous studies have reported the role of auxin in meristem development (Su *et al*. 2011), floral organogenesis and flower fertility (Cheng *et al*. 2006; Brumos *et al*. 2018; Zhang *et al*. 2018; Zhao 2018). It has been proposed that WUS acts as a rheostat in auxin signalling, thereby regulating IM maintenance (Ma *et al*. 2019). Here, MONOPTEROS/AUXIN RESPONSE FACTOR5 (MP/ARF5) is a central TF of the canonical auxin response pathway that translates auxin maxima into development of floral primordia through direct activation of the floral identity gene *LEAFY* (*LFY*) (Yamaguchi *et al*. 2014). In addition, MP activates *AINTEGUMENTA* (*ANT*) and *AINTEGUMENTA‐LIKE*/*PLETHORA* (*AIL6*/*PLT*) genes to promote floral primordia identity and outgrowth (Yamaguchi *et al*. 2013), whereas *ANT* directly binds to and regulates floral organ identity genes, such as class A genes *AP1* and *AP2* and the class E gene *SEP2* (Krizek *et al*. 2020) (Fig. 1). Furthermore, in the non‐canonical auxin response pathway, ETTIN (also known as ARF3) directly binds to the *SHOOT MERISTMLESS* (*STM*) promoter and thereby suppresses its expression to enable FM fate acquisition (Chung *et al*. 2019) (Fig. 1). This is strengthened by MP, which does not control *STM* directly, but activates the expression of *FILAMENTOUS FLOWER* (*FIL*), thereby suppressing *STM* expression (Chung *et al*. 2019). Apart from ETTIN, DNA BINDING WITH ONE FINGER 9 (DOF9), an auxin‐responsive TF from tomato, has recently been reported to control IM and FM differentiation via the regulation of cell division genes and the inflorescence architecture regulator *LYCOPERSICUM INVERTASE*, likely through the ARF5‐DOF9 module (Hu *et al*. 2022). The spatiotemporal fine tuning of auxin concentration is the primary challenge to decipher its regulating mechanism in floral meristem development and floral organogenesis. Moreover, the precise spatiotemporal auxin levels and local biosynthesis within IM and polar transport for FM initiation have been illustrated using classical mutants, such as *PIN*‐*FORMED1* (*PIN1*) (Alvarez‐Buylla *et al*. 2010) encoding a polar auxin carrier (Gälweiler *et al*. 1998). Furthermore, with advanced imaging techniques, it is evident that floral organ initiation at SAMs is dependent on the temporal integration of auxin (Galvan‐Ampudia *et al*. 2020).

In addition to the core auxin signalling components that regulate FM identity and primordia initiation, regulatory mechanisms controlling auxin biosynthesis and distribution further ensure correct reproductive development. The boundary gene *SUPERMAN* (*SUP*) plays a key role in maintaining FM size and coordinating floral organogenesis through fine‐tuning of auxin biosynthesis. SUP interacts with CURLY LEAF (CLF), a histone methyltransferase of the Polycomb Repressive Complex2 (PRC2), to repress the auxin biosynthesis genes *YUCCA1* and *4* (*YUC1/4*), thereby limiting auxin accumulation at the stamen–carpel boundary region (Xu *et al*. 2018) (Fig. 1). Similarly, CRC, a direct target of AG, contributes to the regulation of auxin distribution by repressing the transmembrane protein‐encoding gene *TORNADO2* (*TRN2*), leading to the establishment of auxin maxima required for FM termination and gynoecium formation (Yamaguchi *et al*. 2017) (Fig. 1). The essential roles of auxin across reproductive development, including floral transition, inflorescence formation, floral organogenesis, fruit development and embryogenesis, have been visualized using auxin response sensors (*DR5::VENUS*) and the auxin efflux carrier reporter *AtPIN1::GFP* in *Arabidopsis* and tomato (Aloni *et al*. 2006; Larsson *et al*. 2013, 2014; Goldental‐Cohen *et al*. 2017). In both *Arabidopsis* and rice, PINOID (serine/threonine protein kinase) interacts with PINs to regulate floral organ development through modulation of auxin polar transport (Benjamins *et al*. 2001; Wu *et al*. 2020). Additionally, it has been proposed that PINOID also interacts with OsMADS16, a homologue of *Arabidopsis* AP3, and transcriptionally regulates floral organ development in rice (Wu *et al*. 2020).

## GIBBERELLIC ACID (GA) IN FLORAL REGULATORY NETWORKS AND FLOWERING TIME CONTROL

**Fig. 2:**
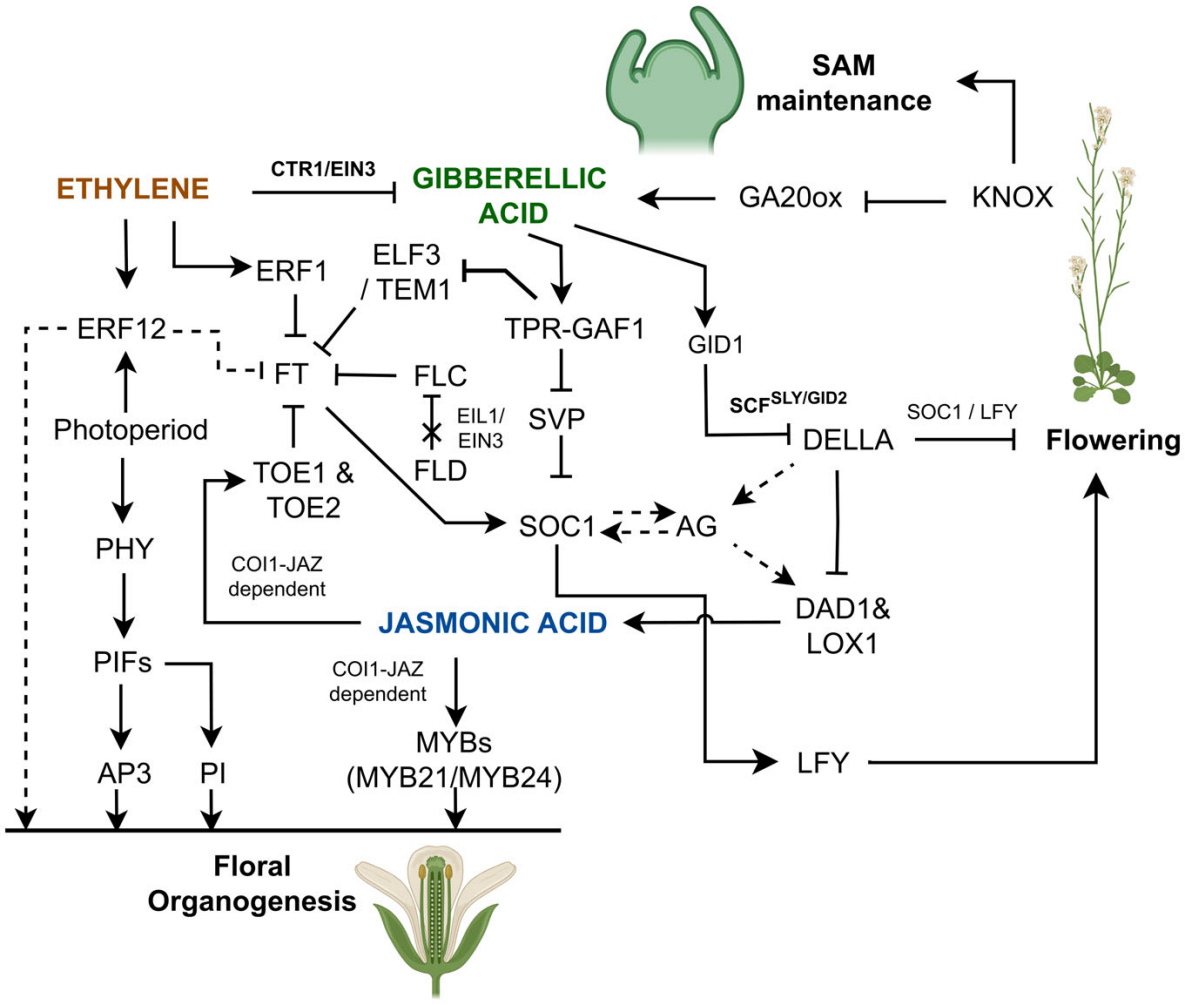
Interplay between gibberellic acid (GA), ethylene and jasmonic acid (JA) in terms of their roles in flower development. ETHYLENE RESPONSE FACTOR (ERF), such as ERF1, negatively regulates floral transition through repression of *FLOWERING LOCUS T* (*FT*), which activates the central floral integrator *SUPPRESSOR OF OVEREXPRESSION OF CONTANS1* (*SOC1*). Photoperiod induces floral organ identity genes *APETELA3* (*AP3*) and *PISTILLATA* (*PI*) through activation of PHYTOCHROME (PHY) INTERACTING FACTOR (PIFs). Furthermore, photoperiod‐dependent induction of TF ERF12 has been reported to regulate floral organogenesis positively, but delays floral transition, which is most likely repressing expression of *FT*. Ethylene signalling TFs, ETHYLENE INSENSITIVE3 (EIN3) and its homologue EIN3 LIKE1 (EIL1), physically associate with FLOWERING LOCUS D (FLD) and inhibit flowering through transcriptional activation of floral repressor *FLOWERING LOCUS C* (*FLC*). Ethylene production enhances ethylene signalling by inhibiting its negative regulator CONSTITUTIVE TRIPLE RESPONSE1 (CTR1) and increasing its master transcriptional positive regulator EIN3. The antagonistic effect of activated ethylene signalling on bioactive GA is modulated via CTR1/EIN3‐dependent ethylene response pathway. In response to GAs, GIBBERELLIN INSENSITIVE ASSOCIATED FACTOR1 (GAF1) interacts with TOPELESS‐RELATED (TPR) and forms a co‐repressor complex to induce expression of *FT* and *SOC1* by repressing flowering negative regulators such as SHORT VEGETATIVE PHASE (SVP), EARLY FLOWERING3 (ELF3), and TEMPRANILLO1 (TEM1). The KNOTTED‐LIKE HOMEOBOX (KNOX) maintains the fate of SAM and creates link with the GA biosynthesis pathway through repression of GA biosynthetic gene *GA20‐OXIDASE*. The binding of GA to its receptor GA INSENSITVE DWARF1 (GID1) enhances the interaction between GID1 and DELLA repressor proteins, resulting in rapid degradation of DELLA through requirement for specific ubiquitin E3 ligase complex (SCF^SLY1/GID2^) for polyubiquitination and subsequent degradation by the 26S proteasome. DELLA proteins repress expression of the floral central regulators *SOC1* and *LFY*, thereby inhibiting floral transition. Furthermore, DELLA supresses expression of JA biosynthetic genes, such as *DEFECTIVE IN ANTHER DEHISCENCE1* (*DAD1*) and *LIPOXYGENASE* (*LOX*), connecting the flowering network between GA and JA. JA negatively regulates flowering through repression of *FT*. The expression of *FT* is supressed by AP2 TFs TARGET OF EAT (TOE1 and TOE2), which are liberated through JA receptor CORONATINE INSENSITIVE1 (COI1)‐dependent degradation of the jasmonate‐ZIM domain (JAZ) repressor. Similarly, JA promotes filament elongation through MYB21/24, elucidating its role in floral organogenesis. Activation is indicated with a ‘sharp arrow’ (→), while inhibition indicated with a ‘blunt arrow’ (┴), and regulation restriction is depicted with cross (✖). Broken lines indicate indirect manipulation of a process or unknown mechanism. Images were created with BioRender.com.

In *Arabidopsis*, the KNOTTED1‐LIKE HOMEOBOX (KNOX) TFs play a pivotal role in maintaining SAM activity by modulating the hormonal balance between CK and GA. They achieve this by promoting CK biosynthesis to facilitate cell division while simultaneously repressing GA biosynthesis, which is crucial for cell elongation and differentiation (Jasinski *et al*. 2005). Recently, a global CHIP‐seq analysis in *Arabidopsis* has identified targets of the STM TF (member of KNOX family) and showed that transcriptional regulators involved in meristem maintenance and hormone signalling are highly enriched. These findings underscore the critical role of KNOX TFs in bridging hormone perception and meristem development within the central regulatory network (Lechon *et al*. 2025). Furthermore, in maize, KNOTTED1 (KN1) enhances expression of *GA2ox1* encoding the GA‐deactivating enzyme GA2‐oxidase, thereby limiting GA levels (Bolduc & Hake 2009). A similar observation was reported for potato, where overexpression of the KNOX‐I gene (*Potato Homeobox 15*) led to elevated transcript levels of *StGA2ox1*, supporting the role of KNOX in regulating GA metabolism (Mahajan *et al*. 2016). In tobacco (*Nicotiana tabacum*), the KNOX homeodomain protein (*Nt*H15) directly supresses the GA biosynthetic gene *GA20‐OXIDASE* (referred to as *Ntc12*) to maintain indeterminacy state of corpus cells of SAM, underpinning the role of the GA‐KNOX module in SAM maintenance (Sakamoto *et al*. 2001) (Fig. 2). Likewise, in rice, elevated expression of *GNP1*, which encodes a GA20‐oxidase, activates a KNOX‐mediated feedback loop that upregulates *GA20ox* expression, resulting in reduced levels of the bioactive gibberellins GA1 and GA3 (Wu *et al*. 2016). Apart from CK and auxin, GA regulates flowering *via* DELLA‐dependent and independent pathways (Mutasa‐Göttgens & Hedden 2009; Ito *et al*. 2018). DELLAs are negative regulators in GA signalling and are degraded upon increase in GA, hence allowing expression of GA‐responsive genes. In *Arabidopsis*, DELLAs interact with PHYTOCHROME INTERACTING FACTORS (PIFs) to modulate photoperiodic flowering (de Lucas *et al*. 2008; Feng *et al*. 2008). The degradation of PIFs and DELLAs through GA signalling (Li *et al*. 2016) partly promotes expression of floral homeotic genes, such as *AP3*, *PI* and *AG* (Yu *et al*. 2004) (Fig. 2). In addition, DELLAs act as coactivators of GIBBERELLIN‐INSENSITIVE ASSOCIATED FACTOR1 (GAF1), which regulates genes encoding GA biosynthetic enzymes (Fukazawa *et al*. 2014). GAF1 functions in the GA‐dependent flowering pathway by regulating expression of *FLOWERING LOCUS T* (*FT*), and *SUPPRESSOR OF OVEREXPRESSION OF CONSTANS1* (*SOC1*) in *Arabidopsis*, through repression of the flowering repressor genes, *EARLY FLOWERING3* (*ELF3*), *SHORT VEGETATIVE PHASE* (*SVP*) and *TEMPRANILLO1* and *2* (*TEM1* and *TEM2*) (Fukazawa *et al*. 2021) (Fig. 2). Under short‐day conditions (SD), GA promotes flowering in *Arabidopsis* through activation of the central floral integrator *SOC1* in the inflorescence (Bonhomme *et al*. 2000; Moon *et al*. 2003) and *LFY* in the floral meristem *via* a DELLA‐independent pathway (Blázquez *et al*. 1998) (Fig. 2). Meanwhile, under inductive long days (LD), GAs are essential in vascular tissue to elevate the transcript level of *FT* and *TWIN SISTER OF FT* (*TSF*), that encode a systemic signal (florigen) transported from leaves to the meristem to induce flowering. GAs are not required to activate *SOC1* under LDs at the SAM, as reported under SDs, but they enhance subsequent steps after floral induction, including activation of genes encoding SQUAMOSA PROMOTER BINDING PROTEIN‐LIKE (SPL or SBP) TFs (Porri *et al*. 2012). Moreover, the FLOWERING PROMOTING FACTOR1 (FPF1) has been implicated in a GA‐dependent floral transition in *Arabidopsis*. FPF1 is a small 12.6 kDa protein that is expressed early during the floral transition in apical meristems and later in floral meristems (Kania *et al*. 1997; Melzer *et al*. 1999). Constitutive expression of *FPF1* caused early flowering both in SD and LD conditions, and genetic and molecular studies showed that *FPF1* synergistically interacts with *LFY* and *AP1* on flowering time regulation. Reducing endogenous GA levels – either by using GA biosynthesis mutants such as *ga1‐3* or through treatment with paclobutrazol, an inhibitor of gibberellin biosynthesis – suppressed the early flowering phenotype induced by *FPF1* overexpression, indicating that *FPF1* promotes flowering through GA signalling (Kania *et al*. 1997; Melzer *et al*. 1999). Furthermore, GAs regulate *Arabidopsis* flowering through direct interaction of DELLA proteins with the age‐dependent miR156‐targeted *SPL* genes, such as *SPL9*, which results in attenuation of its transcriptional activity and, consequently, delays the floral transition by repressing *miR172b* and *SOC1* (Yu *et al*. 2012). Conversely, DELLA proteins such as RGA (REPRESSOR OF GA1‐3) recruited *via* SPL9 to the *AP1* locus induce expression of *AP1* and enhance conversion of lateral primordia into flowers (Yamaguchi, Winter, *et al*. 2014). The complex interaction of GA not only with the miR156‐SPL module in *Arabidopsis* (Galvão *et al*. 2012; Yu *et al*. 2012) but also with tomato miR156‐targeted *SQUAMOSA PROMOTER BINDING PROTEIN–LIKE* (*SlSBPs*) and the miR319 targeted *TEOSINTE BRANCHED1/CYCLOIDEA/PCF* (*TCP*) genes during floral transition has been revealed (Burko *et al*. 2013). In tomato, PROCERA – a DELLA protein – activates expression of floral integrators *SINGLE FLOWER TRUSS* (*SFT*, the tomato orthologue of *FT* and *AP1/MACROCALYX*), where it is repressed by miR319‐targeted *LANCEOLATE* (*LA*) gene, a *TCP4* homologue (Ori *et al*. 2007; Parapunova *et al*. 2014). It has been suggested that *SFT* and floral identity genes are regulated through recruitment of miR156‐targeted *SlSBPs* and their interaction with GA. Furthermore, miR319‐LA module has been shown to integrate with GA for meristem maturation and floral transition in cultivated tomato in a PROCERA‐dependent manner (Silva *et al*. 2019; Ferigolo *et al*. 2023). In addition to meristem maturation and floral transition, GA also plays a crucial role in floral organ development and fertility. In *Arabidopsis*, GA regulates MYB21 and MYB24 by inactivating DELLA proteins (RGA and RGL), suggesting that MYB21 and MYB24 are essential for GA‐mediated stamen development in *Arabidopsis* (Cheng *et al*. 2009).

## ETHYLENE AND ABSCISIC ACID (ABA) IN FLOWERING

The effect of ethylene on flowering predominantly depends on the GA pathway (Achard *et al*. 2007). Ethylene‐dependent regulation of flowering occurs through the reduction of bioactive GAs, and results in an increased accumulation of DELLA proteins that ultimately lead to suppression of the floral inducers *SOC1* and *LFY*. ETHYLENE RESPONSE FACTOR12 (ERF12), a key component of ethylene signalling belonging to the AP2/ERF TF family, and an *Arabidopsis* orthologue of *MULTIFLORET SPIKELET1* (*MFS1*) in rice, affects pleiotropic features of floral development in LDs, including floral organ phyllotaxy, floral organ number in the early‐formed flowers, particularly sepal number, and delays the floral transition under SDs and LDs (Chandler & Werr 2020). Recently, it was shown that ERF1 negatively regulates floral initiation in *Arabidopsis* through direct repression of *FT* transcription and prolongs the vegetative phase during development (Chen *et al*. 2021) (Fig. 2). The core TFs of the ethylene signalling pathway, such as ETHYLENE INSENSITIVE3 (EIN3) and its homologue EIN3 LIKE1 (EIL1), negatively regulate floral transition in *Arabidopsis* by directly binding to *FLC*, modulating its expression through the recruitment of *FLOWERING LOCUS D* (*FLD*) (Xu *et al*. 2021) (Fig. 2). *FLD* encodes a histone demethylase and acts in the autonomous flowering pathway in *Arabidopsis*, which has been shown to supress the floral repressor *FLC* through direct binding and removing di‐methylation marks at Lysine 4 on Histone 3 (He *et al*. 2003; Jiang *et al*. 2007). Apart from the floral transition, ethylene plays a significant role in floral organogenesis in tomato, likely through the HD‐Zip homeobox gene *SlHB*‐*1* that positively controls the key ethylene biosynthetic gene 1‐*AMINOCYCLOPROPANE‐1‐CARBOXYLIC ACID* (*ACC*) *OXIDASE1* (*SlACO1*). A study showed that ectopic overexpression of *SlHB‐1* in tomato increases *SlACO1* transcripts and alters floral organ morphology, including production of multiple flowers or carpel‐like structures within one sepal whorl, fusion of sepals and petals and swelling of the base of sepals (Lin *et al*. 2008). Similarly, ectopic expression of *CsACO2*, a gene from *Cucumis sativus* driven by an *AP3* promoter, caused ethylene overproduction, which resulted in stamen abortion and male sterility in *Arabidopsis* (Duan *et al*. 2008). Although the data suggest the role of ethylene in floral organogenesis through binding of *Sl*HB‐1 protein to *SlACO1* promoter, there is still a lack of evidence underpinning an interconnected regulatory network of ethylene biosynthesis and/or signalling with apical meristem and/or floral meristem development regulators. The lack of clarity of ethylene signalling through ERFs further leads to ambiguity in the inference of its role in floral organogenesis, *viz., ERF19* is not involved in ethylene signalling, but promotes floral meristem activity and flower organ size through regulation of the CLV‐WUS module and auxin signalling pathway, respectively (Lee *et al*. 2023).

**Fig. 3:**
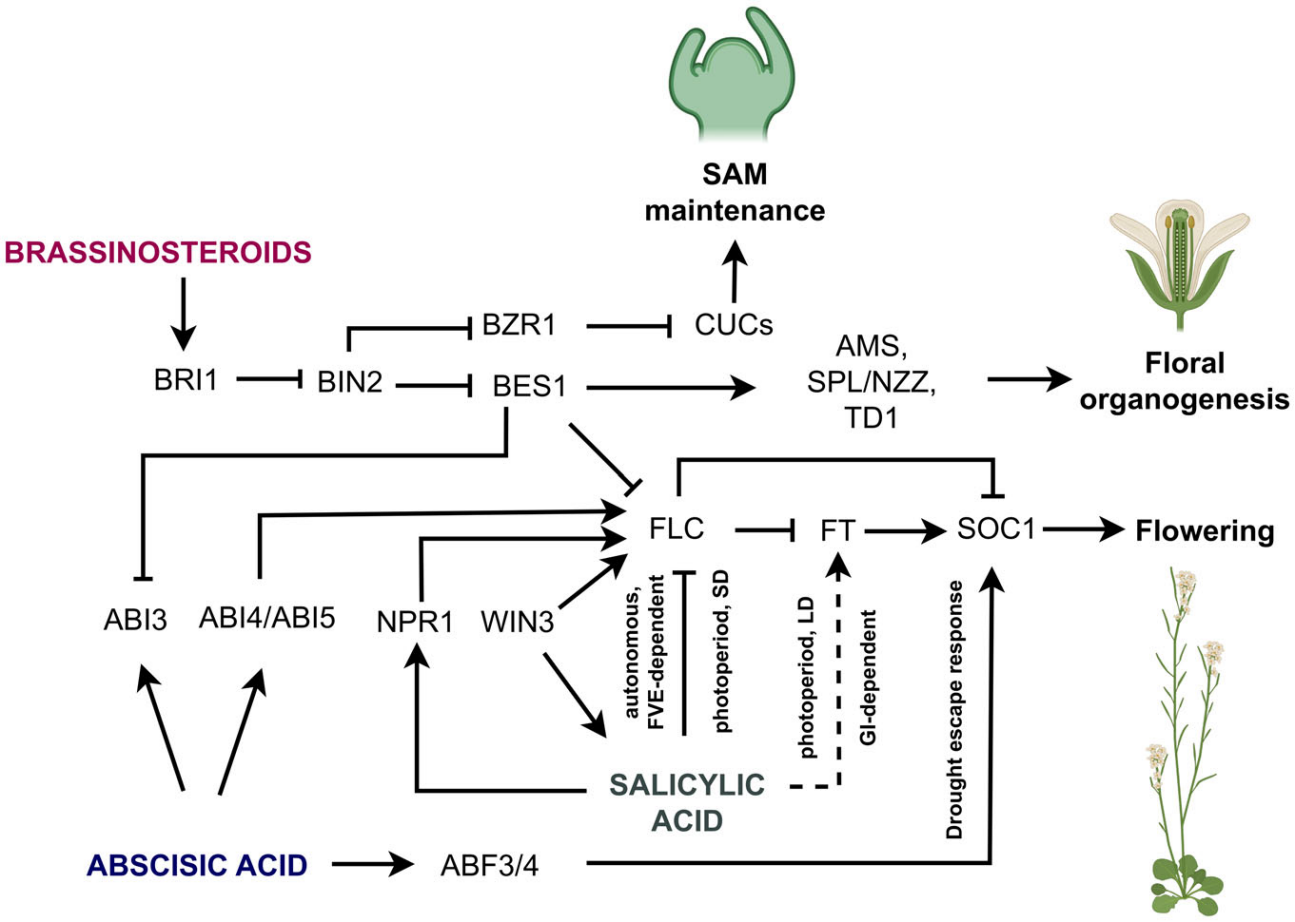
Crosstalk between brassinosteroids (BR), abscisic acid (ABA) and salicylic acid (SA) in terms of their roles in flower development. The BR signalling component BRI1‐EMS‐SUPPRESSOR1 (BES1) is inactivated by negative regulator of BR signalling; BRASSINOSTEROID‐INSENSITIVE2 (BIN2). BRASSINAZOLE RESISTANT1 (BZR1), a BR signalling component directly supresses the *CUP‐SHAPED COTYLEDON* (*CUC*) family of organ boundary identity genes and maintains SAM. BES1 promotes flowering through repression of floral repressor *FLC*. Furthermore, BES1 mediates modulation of key genes for anther and pollen development, such as *SPOROCYTELESS/NOZZLE* (*SPL*/*NZZ*), *DEFECTIVE IN TAPETAL DEVELOPMENT AND FUNCTION1* (*TDF1*) and *ABORTED MICROSPORE* (*AMS*). Additionally, antagonistic crosstalk between ABA and BR is suggested to be mediated through BES1‐dependent suppression of negative regulator of flowering; ABA INSENSENTIVE3 (ABI3) to promote early flowering. ABA stimulated TFs ABI4 and ABI5 activates *FLC* to repress flowering, in contrast ABA‐responsive element (ABRE)‐binding factors (ABF3 and ABF4) promote flowering in response to drought escape. SA promotes flowering through repression of *FLC* in autonomous‐ and photoperiod‐ (SD) dependent flowering pathway. Furthermore, SA activates *FT* through GIGANTEA (GI)‐dependent photoperiod under LD conditions. Additionally, type II SA regulator *HOPW1‐1‐INTERACTING3* (*WIN3*) and a bonafide SA receptor *NONEXPRESSER OF PATHOGENESIS‐RELATED* (*NPR*) negatively influence floral transition through the modulation of *FLC*. Activation is indicated with a ‘sharp arrow’ (→), while inhibition indicated with a ‘blunt arrow’ (┴). Broken lines indicate indirect manipulation of a process or unknown mechanism. Images were created with BioRender.com.

The role of ABA in the floral transition is controversial, since positive and negative effects of ABA have been reported (Domagalska *et al*. 2010; Riboni *et al*. 2013; Conti *et al*. 2014; Martignago *et al*. 2020). The antagonistic roles of ABA in *Arabidopsis* are linked to direct activation of *FLC* transcription, mediated *via* ABA‐stimulated bZIP TF ABSCISIC ACID‐INSENSITIVE4 (ABI4) and AP2/ERF TF ABSCISIC ACID‐INSENSITIVE5 (ABI5) (Wang *et al*. 2013; Shu *et al*. 2018) (Fig. 3). The splicing factor *At*U2AF65b in *Arabidopsis* has been shown to delay flowering time by imparing the pre‐mRNA splicing of *FLC* and *ABI5* in the shoot apex, indicating its role in ABA‐mediated regulation of floral transition (Xiong *et al*. 2019). Moreover, the ABA responsive TF *Sl*MYB55 in tomato was reported to directly target the *WUS* gene and also interacts *in vitro* with the MADS‐box protein *Sl*MBP21, an inhibitor of cell division that negatively regulates the floral transition as well as number and size of flowers. Exogenous application of ABA represses expression of positive flowering regulators *EARLY HEADING DATE* (*Ehd1* and *Ehd2*) in rice. ABA‐responsive element (ABRE)‐binding factor (ABF1) facilitates this repression by recruiting the PRC2 complex, which deposits the repressive histone modification H3K27me3 on the promoters of *Ehd1* and *Ehd2*, thereby reducing their transcription and delaying flowering (Tang *et al*. 2024). In contrast, ABA has been reported as a flowering activator, especially in the drought‐escape (DE) response, *via* the activation of *SOC1* by ABF3 and ABF4 (Hwang *et al*. 2019) (Fig. 3). These bZIP TFs are considered as master regulators of ABA signalling in response to drought and osmotic stress (Kang *et al*. 2002; Yoshida *et al*. 2010). Furthermore, a positive regulator of ABA signalling, the OPEN STOMATA1 (*Sl*OST1) kinase, interacts with and phosphorylates TF VASCULAR PLANT ONE‐ZINC FINGER1 (*Sl*VOZ1), which directly binds to the *FT* orthologue *SINGLE FLOWER TRUSS* (*SFT*) promoter for positively modulating the floral transition in tomato (Chong *et al*. 2022).

## JASMONIC ACID (JA) ORCHESTRATES FLORAL DEVELOPMENT AND ORGANOGENESIS

The occurrence and putative functions of JA and its derivatives, known as jasmonate(s), in floral development have been well reviewed in the last decade (Wasternack *et al*. 2013). JA acts as a negative factor for flowering through repression of *FT* and other flowering time genes (Zhao *et al*. 2022). In *Arabidopsis*, the JA co‐receptor CORONATINE INSENSITIVE1 (COI1)‐dependent degradation of jasmonate‐ZIM domain (JAZ) repressors, liberates the AP2‐like TFs TARGET OF EAT (TOE1 and TOE2) to repress expression of *FT* and thereby delay flowering (Zhai *et al*. 2015) (Fig. 2). The overexpression of *SlJAZ2* in tomato accelerates the floral transition through downregulation of its potential targets *AP2a, AP2b* and *AP2e* (Yu *et al*. 2018), while overexpressing *AtJAZ1* in *Arabidopsis* does not alter flowering time (Zhai *et al*. 2015). The difference in the functions of JAZ proteins in various species increases the complexity of COI1‐dependent JA regulation of flowering time. In addition to the floral transition, JA functions in stamen development, such as filament elongation, as well as anther development and dehiscence (Sanders *et al*. 2000; Ishiguro *et al*. 2001). Recently, a role for JA in floral organogenesis in *Arabidopsis* at an epigenetic level has been shown. The protein enriched with AT‐rich interacting domain (ARID5), a conserved DNA‐binding domain, interacts with the chromatin remodelling complex Imitation of Switch (ISWI) to enhance stamen filament elongation through activation of several JA biosynthesis genes (Zhao *et al*. 2021). It has been shown that the floral homeotic gene *AG* directly regulates expression of *DEFECTIVE IN ANTHER DEHISCENCE1* (*DAD1*), encoding one of the early enzymes of JA biosynthesis, to control late stamen development (Ito *et al*. 2007) (Fig. 2). Additionally, *DAD1* expression in *Arabidopsis* in developing floral organs is activated by *ARF6* and *ARF8* for JA‐dependent flower opening and anther dehiscence (Tabata *et al*. 2010). In *Arabidopsis* and tomato, JA affects floral organ formation through transcriptional regulation mediated by *At*MYB21/24/57/108 and *Sl*MYB21, respectively (Cheng *et al*. 2009; Mandaokar & Browse 2009; Niwa *et al*. 2018). Furthermore, in *Arabidopsis*, JA regulates anther filament elongation through a COI1‐dependent activation of TFs *MYB21*, *MYB24*, *MYB57* and *MYB108*, while in tomato, the homologous regulatory module regulates carpel development, indicating a conserved yet functionally diversified JA signalling pathway across eudicots (Qi *et al*. 2015; Schubert *et al*. 2019; Huang *et al*. 2020) (Fig. 2). Schubert *et al*. (2019) further showed that JA induces expression of *SlMYB21* in tomato to coordinate floral organogenesis and flower opening through a yet unknown mechanism (Fig. 2). In maize, JA also plays a key role in regulating gametophytic organ development. Specifically, JA is required for expression of the *TASSELSEED* gene, which encodes a 13‐LIPOXYGENASE (13‐LOX) involved in JA biosynthesis (Acosta *et al*. 2009). This JA‐dependent signalling pathway mediates pistil abortion in staminate florets, highlighting a conserved role for JA in male floral development across monocots and dicots (Acosta *et al*. 2009). In addition to stamen and carpel organogenesis, JAs are involved in cell growth in petals of *Arabidopsis*, which is controlled by a bHLH TF named BIGPETALp (BPEp) (Brioudes *et al*. 2009). Beyond flower development, JA plays substantial role in regulating the transition from juvenile to adult phase in rice. A loss‐of‐function mutation in the gene *PRECOCIOUS*, which encodes the key biosynthetic enzyme ALLENE OXIDE SYNTHASE (OsAOS1), leads to early phase transition, underpinning JA's role in development phase regulation (Hibara *et al*. 2016).

## SALICYLIC ACID (SA) IN FLORAL DEVELOPMENT AND FLOWERING TIME REGULATION

There is some evidence that SA influences floral organogenesis and flowering (Fig. 3) (Luo *et al*. 2022), however, the underlying mechanism is still unknown. It has been proposed that SA regulates flowering time in *Arabidopsis* through at least three distinct pathways (Martínez *et al*. 2004): (i) a *CONSTANS*‐ (*CO*) independent and *GIGANTEA‐* (*GI*) dependent photoperiodic pathway that may involve *CO*‐like genes under inductive LD conditions; (ii) a photoperiod‐independent *FLOWERING LOCUS C* (*FLC*)‐dependent pathway through repression of the floral repressor *FLC* under SD conditions; and (iii) *FLC*‐independent autonomous pathway, potentially involving *FLOWERING LOCUS VE* (*FVE*) (Fig. 3). Interestingly, exogenous application of SA in *Arabidopsis* alleviated high temperature‐induced reduction in pollen viability and floret fertility through an unknown mechanism (Zhao *et al*. 2018). Using a transcriptomics approach, the gene *PATHOGEN AND CIRCIDIAN CONTROLLED 1* (*PCC1*) has been identified as a potential candidate of SA‐dependent activation of flowering in *Arabidopsis* (Segarra *et al*. 2010). The late‐flowering phenotype of knock‐down *ppc1* plants under LD conditions supports this role in non‐stressed plants through a photoperiod‐dependent pathway, most likely upstream of *FT*, since *FT* is massively downregulated in these mutants (Segarra *et al*. 2010). Furthermore, the SA regulatory gene *HOPW1‐1‐INTERACTING 3* (*WIN3*) negatively influences the floral transition through modulation of *FLC* and *FT*, but independent of *CO* and *SOC1* (Wang *et al*. 2011) (Fig. 3). Similarly, the bonafide SA receptor NPR1 (*NONEXPRESSOR OF PATHOGENESIS‐RELATED GENE1*) negatively influences floral transition through activation of *FLC* (Wang *et al*. 2011; Luo *et al*. 2022) (Fig. 3). In addition, overexpression of *HEAVY METAL‐ASSOCIATED ISOPRENYLATED PLANT PROTEIN3* (*HIPP3*), which encodes a zinc‐binding nuclear protein acting upstream of the SA pathway, results in delayed flowering in *Arabidopsis*, suggesting its involvement in flowering time regulation (Zschiesche *et al*. 2015).

## BRASSINOSTEROIDS (BRs) IN INFLORESCENCE MERISTEM FORMATION, FLORAL TRANSITION AND FLOWERING TIME REGULATION

The BRs are critical for IM formation and the floral transition (Li & He 2020). The molecular components of BR signalling have been extensively studied using mutants with defective BR perception or response (Vert *et al*. 2005; Gendron & Wang 2007; Zhu *et al*. 2013). The BR signalling component BRI1‐EMS‐SUPPRESSOR1 (BES1) is a TF that is inactivated by BRASSINOSTEROID‐INSENSITIVE2 (BIN2), a core negative regulator of BR signalling, and interacts with EARLY FLOWERING6 (ELF6) (Yu *et al*. 2008), a repressor of the photoperiodic flowering pathway. Furthermore, BES1 also interacts with an ELF6‐related protein, RELATIVE OF EARLY FLOWERING6 (REF6), which is suppresses *FLC* leading to early flowering in *Arabidopsis* (Noh *et al*. 2004). FRIGIDA is one of the key positive regulators of *FLC*, which stimulates *FLC* expression through a transcription activation complex (FRI‐C), which subsequently inhibits flowering (Choi *et al*. 2011). Recently, a study elucidated the role of BR signalling in tomato flowering through the interaction of *Sl*FRL (FRIGIDA LIKE) with *Sl*BIN2 (Khan *et al*. 2022). *SlFRL*‐overexpressing tomato lines flowered earlier and exhibited higher *SlBIN2* transcript levels, indicating that its expression is positively regulated by *SlFRL*. In contrast, overexpression of *SlBIN2* inhibited growth and development and also decreased *SlFRL* mRNA levels (Khan *et al*. 2022). However, the precise molecular mechanism underlying the reciprocal regulation of *SlFRL* and *SlBIN2* transcript levels, as well as their protein interaction during floral development, remain to be comprehensively elucidated. Furthermore, there is some evidence that the BR signalling TFs BRASSINAZOLE RESISTANT1 (BZR1) and BES1‐INTERACTING MYC‐LIKEs (BIMs), have an antagonistic role in the floral transition by promoting expression of *FLC* and its homologues *FLM* and *MADS AFFECTING FLOWERING* (*MAF4* and *MAF5*) (Li *et al*. 2018) (Fig. 3). Moreover, BZR1 directly suppresses organ boundary identity genes of the *CUC* family, suggesting a role for BR in the spatiotemporal control of organ boundary formation and morphogenesis in the SAM (Gendron *et al*. 2012) (Fig. 3). Recently, the role of BR signalling in tomato floral development was further expanded using the BR‐insensitive tomato mutant *altered brassinolide sensitivity1* (*abs1*), which is impaired by a missense mutation in the kinase domain, H1012Y, of the tomato *curl3* locus (Mumtaz *et al*. 2020), a homologue of *Arabidopsis BRASSINOSTEROID INSENSITIVE1* (*BRI1*) (Koka *et al*. 2000). The semidwarf tomato *abs1* mutant exhibits early flowering, changes in flower morphology (low flower number and curly flower organs), impaired male fertility, as well as reduced mRNA levels of floral organ development genes (Mumtaz *et al*. 2022). In *Arabidopsis*, flowering time genes from the vernalization pathway partially respond to *bri1* mutation, while autonomous pathway genes were not altered, indicating that BR signalling acts to repress *FLC* for early flowering (Domagalska *et al*. 2007). Although involvement of BR signals in the floral transition of *Arabidopsis* was reviewed a decade ago, phenotypic characterization of key molecular players in BR signalling still need to be explored (Li, Li, *et al*. 2010; Li, Su, *et al*. 2010). Furthermore, a genome‐wide study of *KNOX* genes in rice revealed that *ORYZA SATIVA HOMEOBOX1* (*OSH1*) induces the BR catabolic genes *CYP734A2*, *CYP734A4*, and *CYP734A6* that regulate shoot meristem function and maintain SAM homeostasis (Tsuda *et al*. 2014). In addition to SAM homeostasis, BRs play a role in regulating male fertility via BES1‐mediated modulation of key genes for anther and pollen development in *Arabidopsis*, e.g. *SPOROCYTELESS*/*NOZZLE* (*SPL*/*NZZ*), *DEFECTIVE IN TAPETAL DEVELOPMENT AND FUNCTION1* (*TDF1*), *ABORTED MICROSPORE* (*AMS*), *MYB103*, *MALE STERILE1* (*MS1*) and *MS2* (Ye *et al*. 2010) (Fig. 3).

## HORMONAL CROSSTALK DURING FLORAL TRANSITION AND DEVELOPMENT

Although a role for different hormones in the regulation of floral transition, organogenesis and flowering time has been shown, there is little evidence for their crosstalk (Figs. 1, 2, 3). The crosstalk between auxin and CK is a prerequisite for reproductive development. The potential upstream sensing mechanism for the activation of *ISOPENTENYLTRANSFERASE* (*IPT*), a key CK biosynthetic gene, and its downstream targeted molecular cascade attributing changes in hormonal crosstalk has recently been reviewed (Nguyen *et al*. 2021). In *Arabidopsis*, auxin activates ETTIN, a suppressor of the CK biosynthetic genes *IPT* and *LOG* and hence contributes to FM termination (Zhang *et al*. 2018). Moreover, CYTOKININ RESPONSE FACTORS (CRFs) are TFs that act downstream of CK perception and transcriptionally control genes encoding PIN auxin transporters at a specific PIN CYTOKININ RESPONSE ELEMENT (PCRE) domain to maintain plant reproductive development (Šimášková *et al*. 2015). In addition to ETTIN‐mediated CK suppression, ETTIN directly binds to auxin, leading to its dissociation from the co‐repressor complex of TOPLESS/TOPLESS‐RELATED proteins (Kuhn *et al*. 2020). As a result, ETTIN targets are derepressed, leading to gene expression necessary for gynoecium development. Moreover, TPL co‐repressors, along with Aux/IAA proteins, bind to ARFs at low auxin levels, resulting in suppression of ARF activity (Fenn & Giovannoni 2021). At high auxin levels, Aux/IAA proteins are ubiquitinated and degraded, and ARFs promote the transcription of target genes, thereby modulating auxin‐driven responses, such as cell division and expansion (Fenn & Giovannoni 2021).

Furthermore, TOPLESS interacts with the BR signalling component BES1, via its ERF‐associated amphiphilic repression motif to control organ boundary formation in the SAM (Espinosa‐Ruiz *et al*. 2017). In *Arabidopsis*, the JA biosynthetic genes *DELAYED IN ANTHER DEHISCENCE1* (*DAD1*) and *LIPOXYGENASE 1* (*LOX1*) have been shown to be induced by auxin through ARF6 and ARF8 (Nagpal *et al*. 2005) and are suppressed through DELLA proteins (Cheng *et al*. 2009; Huang *et al*. 2020), thereby regulating filament elongation. In tomato, *arf8ab* mutants exhibit diminished JA levels and show parthenocarpy (Israeli *et al*. 2023). JA regulates tomato stamen development by preventing a premature rise in ethylene, which controls anther dehiscence and pollen release (Dobritzsch *et al*. 2015), whereas ovule development is controlled by JA via crosstalk with auxin and GA, through the involvement of MYB21 (Schubert *et al*. 2019). Apart from JA–GA crosstalk, ethylene interacts with GA through the CTR1 and EIN3 proteins. In *Arabidopsis*, ethylene delays floral transition *via* DELLA‐dependent repression of the floral activator genes *LFY* and *SOC1* (Achard *et al*. 2007). In addition, multiple hormonal signals are reported to integrate floral pathways via GA‐regulated DELLA proteins (Conti 2017). Besides ethylene, ABA is also involved in crosstalk with GA, where ABA INSENSITIVE TFs (ABI4 and ABI5) can supress GA biosynthesis and modulate the floral transition in *Arabidopsis* (Shu *et al*. 2016, 2018). One of the ABA signalling TFs, ABI3, acts as a negative regulator of floral transition and reproduction in wild‐type *Arabidopsis* and tomato plants. A study revealed antagonistic crosstalk between ABA and BR through the epigenetic regulation of *ABI3* by the BR signalling component BES1, via the BR‐activated BES1‐TPL‐HISTONE DEACETYLASE19 (HDA19) repressor complex (Hong *et al*. 2019). The ectopic expression of *ABI3* specifically disrupted the early flowering phenotype observed in *bes1‐D* mutants and caused severe late‐flowering phenotypes (Hong *et al*. 2019). This finding was supported by spatiotemporal expression patterns and global transcriptome analysis of *ABI3*‐overexpressing plants, which confirmed the role of ABI3 in negative regulation of floral transition and reproductive development. Furthermore, the loss of function of *ABI3* resulted in early flowering phenotypes under both LD and SD conditions (Hong *et al*. 2019). These results highlight the importance of BES1‐mediated regulation of *ABI3* in the reproductive phase transition. Besides, ABI3, type 2C protein phosphatases (PP2Cs) play a crucial role in ABA signalling by connecting its upstream PYR/PYL/RCAR (PYL) receptors to the downstream subfamily 2 of the sucrose nonfermenting 1‐related kinases (SnRK2s) (Qiu *et al*. 2022). These impact various physiological processes through the dephosphorylation of Ser/Thr residues and negatively regulate ABA signalling (Wang *et al*. 2021). In tomato, *PP2C2* is highly expressed in flowers and is essential for correct floral organ development (Li *et al*. 2024). In *Sl*PP2C2 knockdown tomato lines, the downregulation of *SlMYB21/108* and auxin/IAA signalling genes during stamen and carpel development results in a phenotype similar to that observed in auxin response IAA9 or ARF17 knockdown tomato flowers. Additionally, PP2C2 positively regulates ABA content and negatively regulates IAA content in tomato flowers (Li *et al*. 2024). Previous studies have shown that JA‐responsive, anther−/ovary‐specific *SlMYB21/108* TFs are also ABA‐inducible (Dai *et al*. 2018). Furthermore, *Sl*PP2C2 physically interacts with these two JA‐responsive TFs (*Sl*MYB21 and *Sl*MYB108), as well as with *Sl*FZY (a flavin monooxygenase and YUC homologue from tomato involved in a rate‐limiting step of IAA biosynthesis) and *Sl*SAUR67 (a small auxin upregulated RNA; IAA signalling protein) (Li *et al*. 2024). These findings suggest that *Sl*PP2C2 regulates early floral organ development through crosstalk with *Sl*MYB21/108 and auxin/IAA signalling.

## CONCLUDING REMARKS AND PERSPECTIVES

Flower development relies on the intricate regulation of floral organ identity genes, where progress has been made in connecting hormone pathways to gene hierarchies. However, further identification of floral homeotic gene targets is needed to reveal the specific connections between organs and hormone functions. Hormonal regulation in reproductive organs, particularly during male development, is complex and involves multiple hormones (Chandler 2011). While some interconnections between TF hierarchies and hormones have been elucidated (Herrera‐Ubaldo & de Folter 2022), more research is needed in terms of post‐transcriptional regulation of those TFs. Auxin and GA are pivotal in initiating and orchestrating floral organ development, with redundancy playing a crucial role (Cheng *et al*. 2004; Yu *et al*. 2004; Cucinotta *et al*. 2021). The precise role of auxin gradients in floral development remains unclear, and the redistribution of hormones between developing organs is an open question. In addition to promoting growth, hormones also regulate organ boundaries (Yu & Huang 2016), however how these different types of regulators interact in floral boundary formation is one important question that remains to be analysed. Moreover, specific functions of same hormones have been described for different tissues. In *Arabidopsis* and tomato, JA was found to regulate stamen (Mandaokar *et al*. 2006; Cheng *et al*. 2009; Mandaokar & Browse 2009) and carpel (Li *et al*. 2004; Schubert *et al*. 2019) development, respectively; the molecular mechanism governing species‐dependent organ‐specific development is, however, utterly unknown. Therefore, the extent of hormone cross‐regulation and conservation of floral organ regulation across plant species of varying morphologies are intriguing areas of research.

Coordination within and between different hormone metabolic pathways is one of the most complex regulatory networks in plant physiology, hence the development of new strategies will be required to study the intricate communication(s). Most studies employed to date focus on the molecular regulatory networks governing hormone‐mediated developmental fates, where almost no information is available in terms of the absolute level of hormone metabolites. Although a significant improvement has been done in the field of analytical methods (e.g., through Mass Spectrometry) in the last decade, the major limitation to studying hormone physiology is the presence of only minute, below detection range, quantities of these molecules in designated plant tissues. Hence, an immediate attempt must be made to develop further sensitive tools and methods to detect hormones in a cell‐specific manner, which will eventually help in understanding hormone biology at a deeper and clearer level. Such advances will not only refine our mechanistic understanding of hormone biology but also provide a foundation for translational applications in crop improvement. Leveraging the regulatory roles of hormones in floral initiation and organ specification will provide more possibilities to precisely manipulate flowering time, floral architecture, and reproductive synchrony in major crops, such as rice, maize, wheat and tomato. This offers promising avenues for breeding yield‐stable, resilient cultivars capable of coping with the adverse environmental stresses. Targeted modulation of hormone‐responsive pathways through gene editing or controlled expression will enhance floral development and support climate adaptation.

## AUTHOR CONTRIBUTIONS

RB, AV, SM, BH and SP wrote the manuscript.

## Data Availability

No experimental data are associated with this review.
